# Detection of AMA-M2 in human saliva: Potentials in diagnosis and monitoring of primary biliary cholangitis

**DOI:** 10.1038/s41598-017-00906-1

**Published:** 2017-04-11

**Authors:** Chong Lu, Xianliang Hou, Minwei Li, Lin Wang, Ping Zeng, Hongyu Jia, Jianing Chen, Yingfeng Wei, Hong He, Xiangdong Liu, Hongyan Diao

**Affiliations:** 1 State Key Laboratory for Diagnosis and Treatment of Infectious Diseases, Collaborative Innovation Center for Diagnosis and Treatment of Infectious Diseases, The First Affiliated Hospital, College of Medicine, Zhejiang University, 310003 Hangzhou, China; 2 Affiliated Stomatology Hospital, School of Medicine, Zhejiang University, 310006 Hangzhou, China; 3 Key Laboratory of Advanced Textile Materials and Manufacturing Technology, Ministry of Education, College of Materials and Textile, Zhejiang Sci-Tech University, 310018 Hangzhou, China

## Abstract

Serum anti-mitochondrial antibody type 2 (AMA-M2) is considered as a pivotal biomarker for the diagnosis of primary biliary cholangitis (PBC). However, serological tests have many limitations, including inconvenience, invasiveness, and infection risks. Thus, a less invasive approach to detect AMA-M2 titer is desirable. We examined salivary AMA-M2 of potential PBC patients and found that AMA-M2 could be detected only in saliva of serum AMA-M2-positive PBC patients, but not in saliva of serum AMA-M2-negative PBC patients, oral lichen planus patients (OLP) patients, or healthy controls. Furthermore, the concentration of salivary AMA-M2 was positively correlated with the amount of serum AMA-M2 in patients. The salivary inflammatory cytokines were increased in the PBC, consistent with the results of serum test. These findings indicated that saliva might be a less invasive and cost-effective medium to accurately test for AMA-M2 levels and this is a promising development for the diagnosis and monitoring of PBC.

## Introduction

Saliva is an exocrine secretion from the salivary glands composed of 99% water. It also contains gingival crevicular fluid, serum, and other cellular components, such as proteins, enzymes, antibodies, and cytokines^1, 2^. It was shown that proteins and other substances enter saliva from the blood through passive diffusion or active transport, indicating that many substances found in the blood might also be present in saliva^3^. For example, salivary glucose level was reported to be associated with serum glucose level in healthy individuals^4^. Likewise, a positive correlation of endothelin concentrations was found between saliva and plasma in patients with congestive heart failure^5^. Taken together, saliva could be an effective and much less invasive medium for effectively diagnosing human diseases and monitoring a patient’s health.

Primary biliary cholangitis (PBC) is a chronic autoimmune liver disease associated with progressive destruction of small intrahepatic bile ducts^6^. Similar to other autoimmune diseases, like systemic lupus erythematosus^7^ and rheumatoid arthritis^8^, females are diagnosed more often than males with a frequency of about ten to one^6^. The diagnosis of PBC is typically based on abnormal serum biochemical parameters, such as the presence of anti-mitochondrial antibody (AMA), increased alkaline phosphatase (ALP), and a positive reaction for gamma-glutamyl transferase (GGT)^9^. Serum AMA, especially the AMA-M2 subtype, is regarded as one of the most specific and acceptable diagnostic indicators for PBC^10, 11^. Since the presence of AMA was first recognized by Walker *et al*. in serum samples of PBC patients in 1965^12^, the high titer of AMA has become a serological diagnostic hallmark of PBC with approximately 95% sensitivity^6, 10, 13^. However, the clinical features of PBC are non-specific, which make the early detection and diagnosis is still rather difficult. Anyone with findings of chronic cholestasis or raised concentrations of ALP should be considered a suspected PBC case. When diagnosed early and properly treated, PBC patients generally respond well to medical therapy, on the contrary, others with delayed diagnosis and treatment usually have to undergo liver transplantation^14^. Blood test may be tough to perform especially in suspected patients living in remote villages with poor medical condition or rather away from the hospital. Recently, immense interest has been converted to develop a more convenient, less-invasive diagnostic approach as an alternative to blood test. Salivary test is, therefore, a preferable option once its clinical value has been identified. Currently, the clinical value of this approach has been increasingly highlighted in the literature, suggesting that saliva could be an improved diagnostic medium^15–18^.

Herein, we investigated whether AMA-M2 could be detected in the saliva of PBC patients, and whether salivary AMA-M2 could provide a novel and practical biomarker for precise diagnosis of PBC.

## Results

### AMA-M2 was detectable in saliva of PBC patients

**Figure 1:**
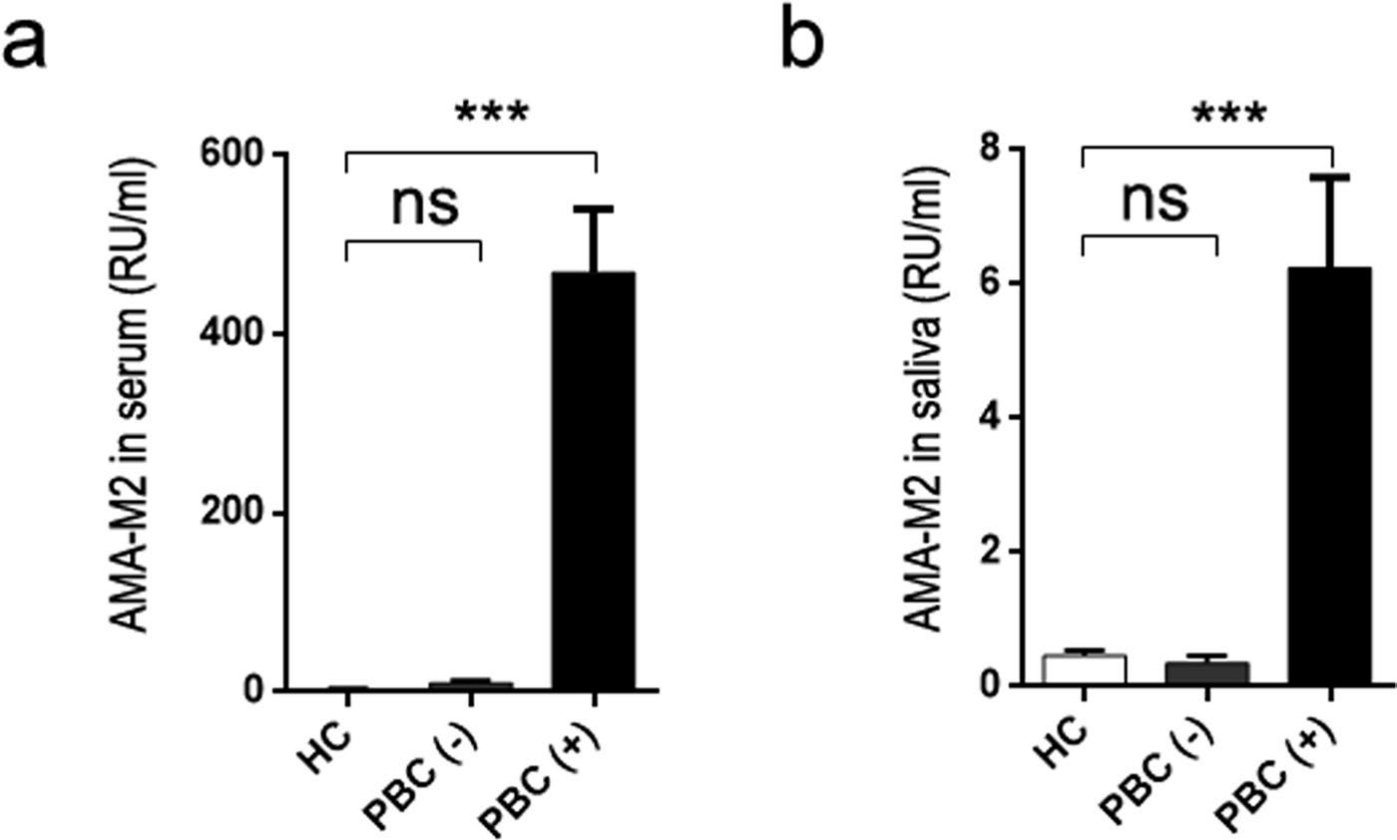
AMA-M2 was detectable in saliva of PBC patients. Levels of anti-mitochondrial antibody subtype M2 (AMA-M2) were measured in collected serum (**a**) and saliva (**b**) of healthy controls (HCs, n = 60), primary biliary cholangitis (PBC) (−) patients (serum AMA-M2-negative PBC patients, n = 16), and PBC (+) patients (serum AMA-M2-positive PBC patients, n = 33). Data presented are the means ± SEM. *P < 0.05; **P < 0.01; ***P < 0.001; ns, not significant.

In this study, we measured the levels of salivary and serum AMA-M2 in all participants. Positive results (i.e. that the value of serum AMA-M2 exceed 40 RU/ml)^9^ for serum AMA-M2 were detected in 33 out of 49 PBC patients (469.20 ± 71.31 RU/ml), while all 60 HC subjects showed negative (3.11 ± 0.43 RU/ml) (Fig. 1a). Importantly, salivary AMA-M2 could be detected only in the serum AMA-M2-positive patients, whereas significant levels could not be detected in any serum AMA-M2-negative patients or HC subjects. Additionally, none of the patients diagnosed with the immune-related mouth disease OLP displayed detectable levels of salivary AMA-M2 (Fig. 1b, Supplementary Figure 1a).

### The level of salivary AMA-M2 was positively associated with the level of serum AMA-M2 in PBC patients

**Figure 2:**
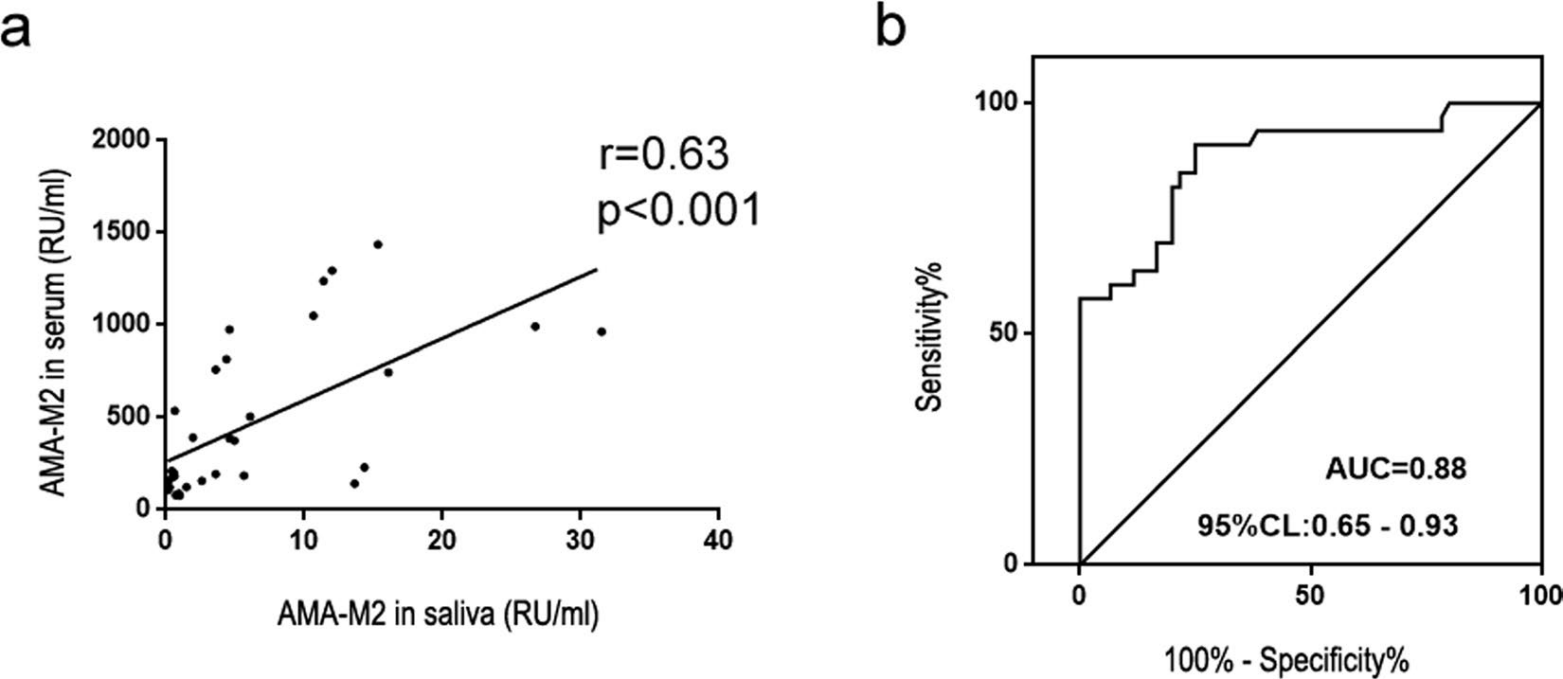
Correlation analysis performed between salivary AMA-M2 and serum AMA-M2 and ROC analysis of salivary AMA-M2 in PBC patients. (**a**) Correlation between salivary AMA-M2 and serum AMA-M2 levels in patients with PBC (serum AMA-M2-positive PBC patients, n = 33). (**b**) Receiver operating characteristics (ROC) curve of salivary AMA-M2 for determining PBC.

We next tested whether there was a correlation between the detectable levels of salivary versus serum AMA-M2. Indeed, salivary AMA-M2 was shown to be significantly positively associated (r = 0.63, P < 0.001) with levels of serum AMA-M2 in PBC patients (Fig. 2a). Furthermore, in order to predict a threshold value of salivary AMA-M2 that could be used for the diagnosis of PBC, ROC was performed. The area under the curve (AUC) was 0.88 (95% CL = 0.65–0.93), and the best diagnostic threshold value was 0.61 RU/ml with a sensitivity of 81.82% and specificity of 80.00% (Fig. 2b).

Furthermore, we collected 10 serum and saliva samples from return visit PBC patients to validate our results. Serum AMA-M2 tests were positive in all the samples, while the levels of salivary AMA-M2 were higher than the cut-off point in 90% of the return visit PBC patients. (Supplementary Figure 2a,b)

Moreover, we analyzed changes in serum and salivary AMA-M2 levels in two collections from the same PBC patients. The variation of salivary AMA-M2 in most of the PBC patients was consistent with that of serum AMA-M2. (Supplementary Figure 3).

### Salivary AMA-M2 does not correlate with other serum hepatic function indices related to cholestasis

**Figure 3:**
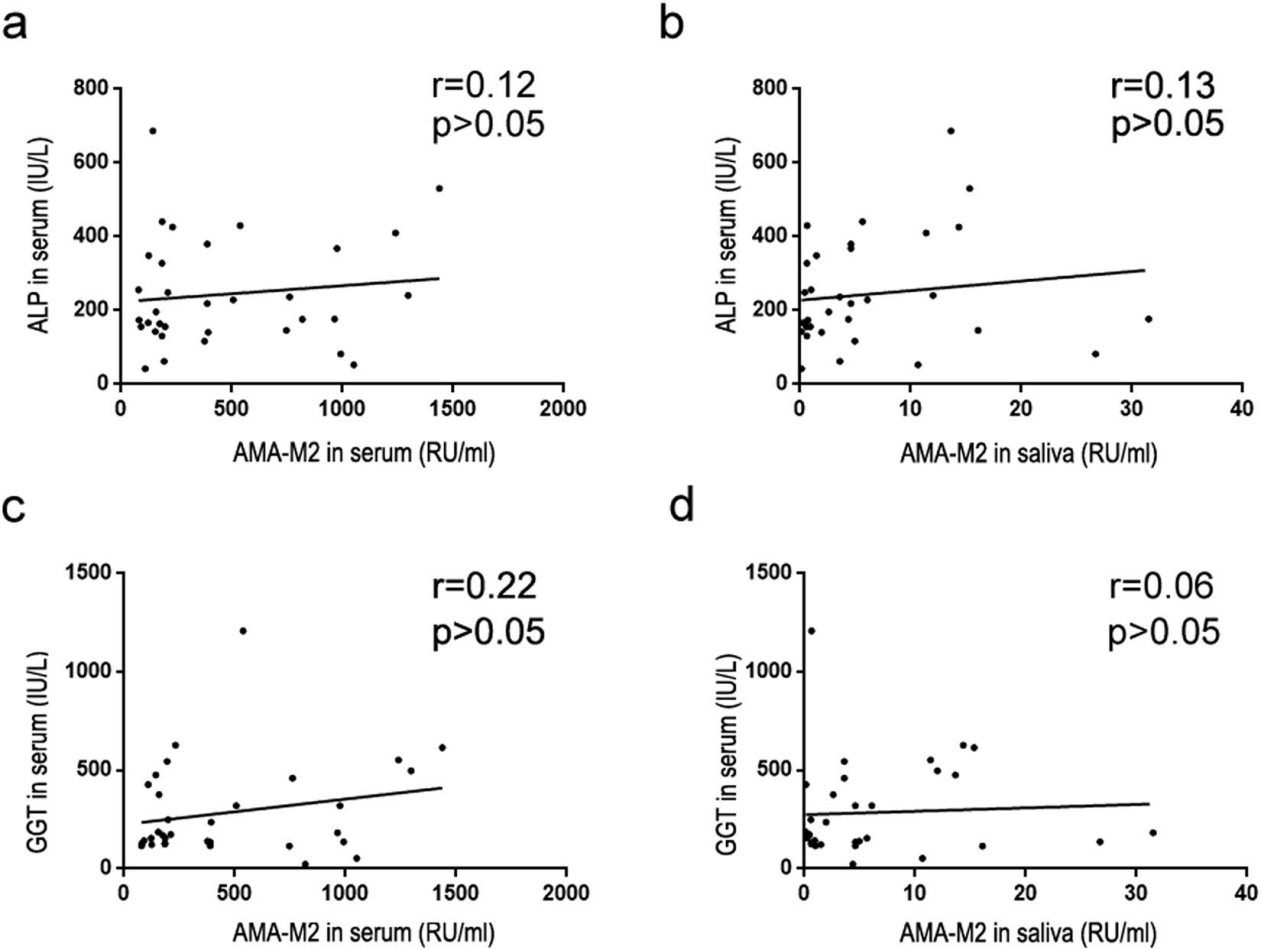
Serum and salivary AMA-M2 levels did not correlate with other hepatic function indicators. (**a**) Correlation between serum AMA-M2 and serum ALP levels in patients with PBC (serum AMA-M2-positive PBC patients, n = 33). (**b**) Correlation between salivary AMA-M2 and serum ALP levels in patients with PBC (serum AMA-M2-positive PBC patients, n = 33). (**c**) Correlation between serum AMA-M2 and serum GGT levels in patients with PBC (serum AMA-M2-positive PBC patients, n = 33). (**d**) Correlation between salivary AMA-M2 and serum GGT levels in patients with PBC (serum AMA-M2-positive PBC patients, n = 33).

Elevated ALP is associated with cholestasis and biliary tract injury. Cholestasis and biliary tract injury present in PBC patients, and these conditions worsen as the disease progresses^9, 19^. Furthermore, elevated GGT is a valuable indicator of bile duct obstruction^20–22^, which often manifests in PBC patients. We tested whether ALP or GGT levels correlated with serum or salivary AMA-M2. However, neither serum ALP levels (Fig. 3a,b) nor serum GGT levels (Fig. 3c,d) were found to correlate with serum or salivary AMA-M2 levels in PBC patients. Taken together, salivary AMA-M2 could be a promising proxy for accurately diagnosing PBC.

### The elevated levels of inflammatory cytokines in saliva reflect a disorder of the oral immune system

**Figure 4:**
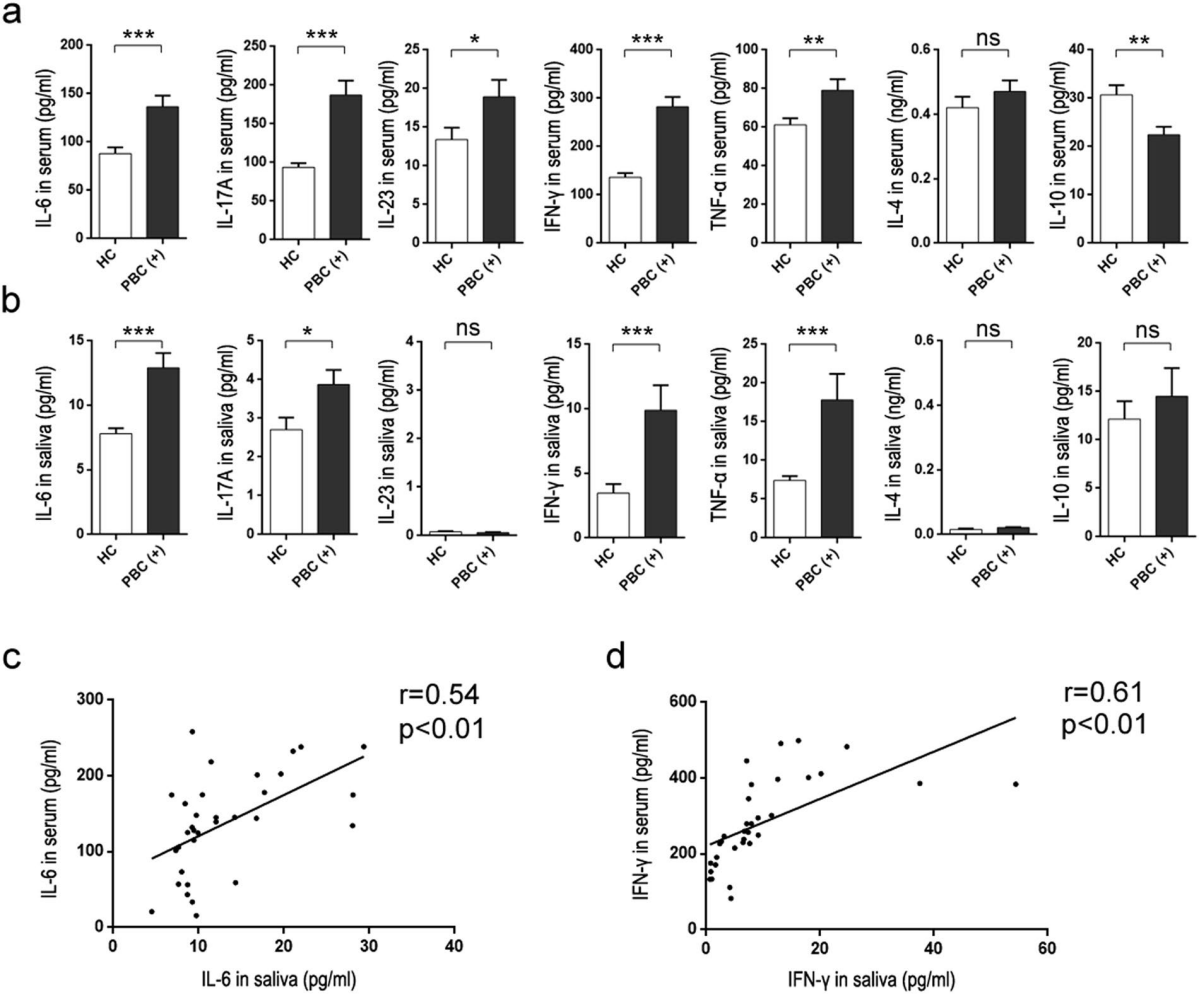
Inflammatory cytokine levels in serum and saliva of PBC patients. The levels of IL-6, IL-17A, IFN-γ, TNF-α, IL-23, IL-4, and IL-10 in serum (**a**) and saliva (**b**) of HCs (healthy controls, n = 60) and PBC patients (serum AMA-M2-positive PBC patients, n = 33) were determined using Luminex Bead Assay. Data presented are the means ± SEM. (**c**) Correlation between salivary IL-6 and serum IL-6 levels in patients with PBC (serum AMA-M2-positive PBC patients, n = 33). (**d**) Correlation between salivary IFN-γ and serum IFN-γ levels in patients with PBC (serum AMA-M2-positive PBC patients, n = 33). *P < 0.05; **P < 0.01; ***P < 0.001; ns, not significant.

Next, we tested the relationship between levels of inflammatory cytokines in the serum versus saliva in PBC patients. Since IL-17A has been reported to play a key role in the inflammatory reaction of PBC patients^23–25^, we examined the levels of inflammatory cytokines associated with IL-17 signaling and immune activation in the serum of all participants. We found that the serum levels of IL-6, IL-17A, IL-23, IFN-γ, and TNF-α in PBC patients were significantly higher compared to HC subjects. Furthermore, the serum immunosuppressive cytokine IL-10 was significantly downregulated in PBC patients (Fig. 4a).

In saliva samples, IL-6 and IFN-γ levels were significantly increased in the PBC group. Meanwhile IL-10, IL-17A, and TNF-α levels were elevated in PBC patients, but these results were not statistically significant (Fig. 4b). IL-4 and IL-23 could not be detected in saliva samples. Furthermore, we found that levels of salivary IL-6, IFN-γ, IL-10, IL-17A, and TNF-α in OLP patients were significantly upregulated, likely because of the oral immune disorder (Supplementary Figure 1b).

To investigate whether saliva inflammatory factors could reflect immune status, we explored the relationship between IL-6 and IFN-γ in saliva and serum. Intriguingly, we discovered that serum IL-6 and IFN-γ levels in PBC patients were positively associated with those in saliva (Fig. 4c,d), but no obvious association was observed for IL-10, IL-17A, or TNF-α in saliva versus serum.

## Discussion

Early and precise diagnosis of PBC is still a great challenge. Therefore, many studies have explored ways to optimize diagnostic methods. Tan and his colleagues proposed that serum microRNA had considerable clinical value in PBC diagnosis with high diagnostic accuracy. However, its high cost limits its clinical application. Salivary AMA-M2 tests may be a more cost-effective and convenient diagnostic method for PBC^26^. Saliva is a particularly tantalizing diagnostic medium, it is easy to collect, store and transport, and these steps do not require highly trained personnel. Patients can even collect samples and complete detection themselves at home, which increases the chances of identifying diseases at early stages and may cost less than blood test by saving the charge of blood collection and processing. Furthermore, saliva collection does not carry with it the risks of infection like serum collection does.

Saliva is already of great clinical value for monitoring a patient’s general health and disease screening for medical problems^27–31^. Numerous studies have demonstrated that saliva could be used to diagnose human diseases. For example, biomarkers found in saliva might be used to screen for breast cancer with high specificity and sensitivity^32^. Cabral *et al*. proposed that salivary cortisol concentration was an excellent indicator of stress of newborns^33^. Likewise, Mascarenhas *et al*. suggested that salivary glucose levels could be a potential biomarker for type 2 diabetes mellitus^34^. It has been speculated that an early factor in the development of PBC is the loss of tolerance to the mitochondrial autoantigen^35, 36^ and the presence of AMA-M2 in serum is a hallmark of PBC.

For these reasons, our study focused on whether saliva might be valuable in diagnosing PBC. In this study, we found that the established biomarker for PBC, AMA-M2, could be detected in saliva. It was only detected at significant concentrations in the saliva of PBC-positive patients, but not HC subjects or OLP patients. Furthermore, salivary and serum AMA-M2 levels were positively correlated, and ROC analysis of salivary AMA-M2 demonstrated its high sensitivity and specificity for making a PBC diagnosis. A salivary AMA-M2 test would be a beneficial tool to screen or monitor groups at high-risk of PBC in the future.

It is reported that AMA-M2 levels are unaffected by treatment^37^. Meanwhile, we examined whether salivary AMA-M2 is related to ALP or GGT, either of which is elevated during the pathogenesis of cholestasis in PBC patients. Surprisingly, salivary AMA-M2 did not correlate with these vital indices of cholestasis. Cumulatively, our results suggested that detection of salivary AMA-M2 is a useful proxy for estimating serum AMA-M2 levels. Even though salivary AMA-M2 is not predictive of factors associated with cholestasis in PBC patients, saliva may still be an excellent alternative to serum for general diagnosis of this disease. Besides, in future, we will try to enlarge our sample size and prolong follow-up time to further clarify whether there exists potential relations between salivary AMA-M2 titer and clinical features.

PBC is a complex autoimmune disease that affects numerous organ systems. It is commonly concomitant with other disorders, including oral disease. Dry mouth and oral candidiasis are always found in PBC patients^6^, in addition to rampant dental caries^38^. This might imply that there is an imbalance in the oral immune environment in PBC patients. Therefore, we examined inflammatory cytokines in the saliva of patients with PBC. We found that salivary IL-6, IFN-γ, IL-17A, and TNF-α were higher in PBC patients compared to HCs. OLP is a chronic inflammatory disease of the oral mucosa. Several researchers have demonstrated that immune dysregulation and complex cytokine networks play important roles in the origin and development of OLP^39–41^. In this study, we similarly observed that salivary inflammatory cytokines were significantly increased in OLP patients. This may reflect similarities in the aberrant oral immune environment of PBC and OLP patients.

To date, current AMA-M2 kits are mostly designed for serum. Since our ROC analysis revealed that AMA-M2 levels in saliva are much lower than in serum, detection methods with higher sensitivity must be developed to achieve the clinical application of AMA-M2 salivary test. Thus, we further applied a new measurement technique called single molecule counting (SMC) technology^42^ to detect salivary AMA-M2. This method demonstrated a higher sensitivity in saliva tests. However, SMC technology is still new, and there are many limitations to practical applications, such as high costs and inconvenient operation. Nonetheless, with the rapid advancements in SMC and other technologies, the detection of salivary AMA-M2 will undoubtedly become easier and more accurate in the future.

In conclusion, our study showed that salivary AMA-M2 has important value for PBC diagnosis, and it might serve as a useful biomarker for this disease. We suggest that this approach may provide a new non-invasive method for the early diagnosis of PBC. In addition, inflammatory cytokine levels in saliva could be used to assess immune disorders of the oral cavity in these patients. A multi-disciplinary cooperative approach should be taken to ensure the wide clinical application of this diagnostic technique.

## Methods

### Ethics statement

Written informed consent was obtained from all the enrolled participants. The study protocol was conducted with the approval of the Ethics Review Committee of the First Affiliated Hospital, Zhejiang University (Permit number: 2016–261), and the use of human blood and saliva samples was in accordance with the Guidelines of the Declaration of Helsinki.

### Subjects and Samples

We recruited 49 patients with PBC for this study, which included 42 females and 7 males. All serum and saliva samples from these patients were obtained from the First Affiliated Hospital at Zhejiang University, and stored at −80 °C for later analysis. The diagnosis of PBC was confirmed by clinical experts based upon the diagnostic criteria proposed by the AASLD^9^. Specifically, in previous clinical examination, immunoserological tests were positive for AMA-M2 in all 49 patients. Additionally, serum ALP levels ranged from 53 to 542. Sixty healthy individuals matched for sex and age were also recruited as controls. Clinical data from the 49 PBC patients and 60 healthy controls (HCs) were collected, including age, gender, and serum levels of aspartate aminotransferase (AST), alanine aminotransferase (ALT), gamma-glutamyl transferase (GGT), alkaline phosphatase (ALP), and total bilirubin (TBIL) (Table 1). In order to explore the immunoenvironment of the oral cavity, saliva samples from 42 patients with oral lichen planus (OLP) matched for gender and age were collected and served as positive controls (Supplementary Table 1). All OLP patients were clinically confirmed, but none displayed systemic disease or received medication.

**Table 1:** Clinical characteristics of the individuals enrolled in the study.

	Healthy controls (60 cases)	PBC patients (49 cases)
Age (years)	50.65 ± 1.40	52.94 ± 1.49
Gender (male/female)	12/48	7/42
AST (IU/L)	24.98 ± 1.21	146.92 ± 11.69
ALT (IU/L)	20.45 ± 0.68	201.41 ± 20.22
GGT (IU/L)	18.23 ± 0.72	268.08 ± 31.19
ALP (IU/L)	23.53 ± 1.06	210.37 ± 14.70
TBIL (umol/L)	31.35 ± 2.16	109.76 ± 10.83

Data are presented as mean ± SEM.

AST, aspartate aminotransferase; ALT, alanine aminotransferase; GGT, gamma-glutamyl transferase; ALP, alkaline phosphatase; TBIL, total bilirubin.

### Measurement of Serum Cholestasis Indices

ALP and GGT were measured using a standard clinical automated analyzer (SRL, Tokyo, Japan).

### Serum and Salivary AMA-M2 Detection

The levels of serum AMA-M2 in PBC patients and HC subjects were determined by enzyme-linked immunosorbent assay (ELISA) (Euroimmun, Germany) according to the manufacturer’s instructions.

To assess the levels of salivary AMA-M2, saliva samples were diluted with 0.1% Bull Serum Albumin-Phosphate Buffer Solution (BSA-PBS) (1:40). Subsequently, two-fold serial dilutions of the top standard from the ELISA kit using 0.1% BSA-PBS were performed to produce a standard curve for a total of eight points. Other experimental procedures on the salivary AMA-M2 test were the same as those performed on serum AMA-M2.

### Luminex Bead Assay

The levels of serum and saliva cytokines were determined using a Bio-Plex Suspension Array System (Bio-Rad) with a commercial 7-plex cytokine (IL-4, IL-6, IL-10, IL-17, IL-23, IFN-γ, TNF-α) detection kit (Millipore, Germany) according to the manufacturer’s protocol.

### Statistical Analysis

Statistical analyses were performed using GraphPad Prism 6 (GraphPad Software, La Jolla, CA, USA). All values were expressed as the mean ± the standard error of the mean (SEM). Generally, differences between two groups were analyzed using the Mann-Whitney U test, and multigroup comparisons were performed by one-way ANOVA. Pearson’s correlation analysis was performed, and scatter plots were drawn to analyze the relationship between two groups. Receiver operating characteristics (ROC) curves were constructed to determine the optimal cut-off value. P < 0.05 was considered statistically significant. *P < 0.05; **P < 0.01; ***P < 0.001; ns, not significant.

## Electronic supplementary material

supplementary material
