# Supplementary material for: Detection of AMA-M2 in human saliva: Potentials in diagnosis and monitoring of primary biliary cholangitis
Source: Sci Rep. 2017 Apr 11;7:796. doi: 10.1038/s41598-017-00906-1 (PMC5429755; doi:10.1038/s41598-017-00906-1)
Supplement: Supplementary file 1 — supplementary material [file 41598_2017_906_MOESM1_ESM.pdf]

Detection of AMA-M2 in human saliva: Potentials in diagnosis and monitoring of primary biliary cholangitis

Author Names

Chong Lu[1], Xianliang Hou[1], Minwei Li[1], Lin Wang[1], Ping Zeng[1], Hongyu Jia[1], Jianing Chen[1], Yingfeng Wei[1], Hong He[2], Xiangdong Liu[3], *Hongyan Diao[1]

[1]State Key Laboratory for Diagnosis and Treatment of Infectious Diseases, Collaborative Innovation Center for Diagnosis and Treatment of Infectious Diseases, The First Affiliated Hospital, College of Medicine, Zhejiang University, 310003 Hangzhou, China

[2]Affiliated Stomatology Hospital, School of Medicine, Zhejiang University, 310006 Hangzhou, China

[3]Key Laboratory of Advanced Textile Materials and Manufacturing Technology, Ministry of Education, College of Materials and Textile, Zhejiang Sci-Tech University, 310018 Hangzhou, China.

* **Correspondence:** Hongyan Diao, State Key Laboratory for Diagnosis and Treatment of Infectious Diseases, The First Affiliated Hospital, School of Medicine, Zhejiang University, Hangzhou, 310003, China. E-mail: diaohy@zju.edu.cn

Supplementary Figure 1

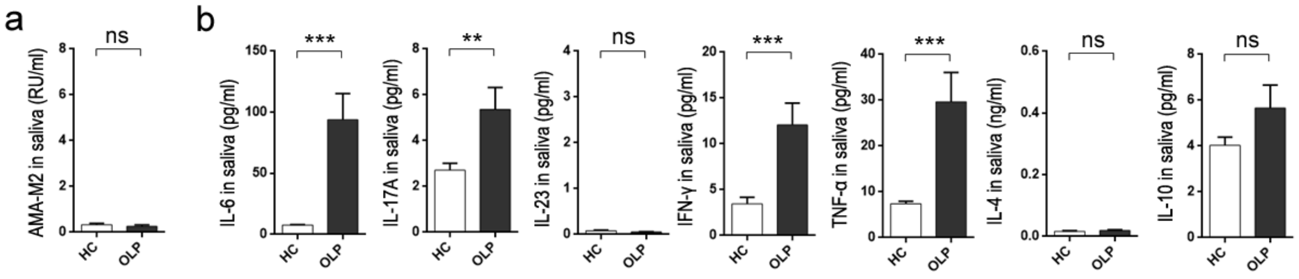

Supplementary Figure 1: AMA-M2 and inflammatory cytokine levels in saliva from OLP patients versus healthy controls

(a) Levels of AMA-M2 were measured in saliva of HCs (healthy controls, n = 60) and OLP patients (n = 42). **(b)** IL-6, IL-17A, IFN-γ, TNF-α, IL-23, IL-4, and IL-10 levels in saliva of HC (healthy controls, n = 60) and OLP patients (n = 42) were determined using Luminex Bead Assay. Data presented are the means ± SEM. *$p < 0.05$, **$p < 0.01$, ***$p < 0.001$.

Supplementary Figure 2

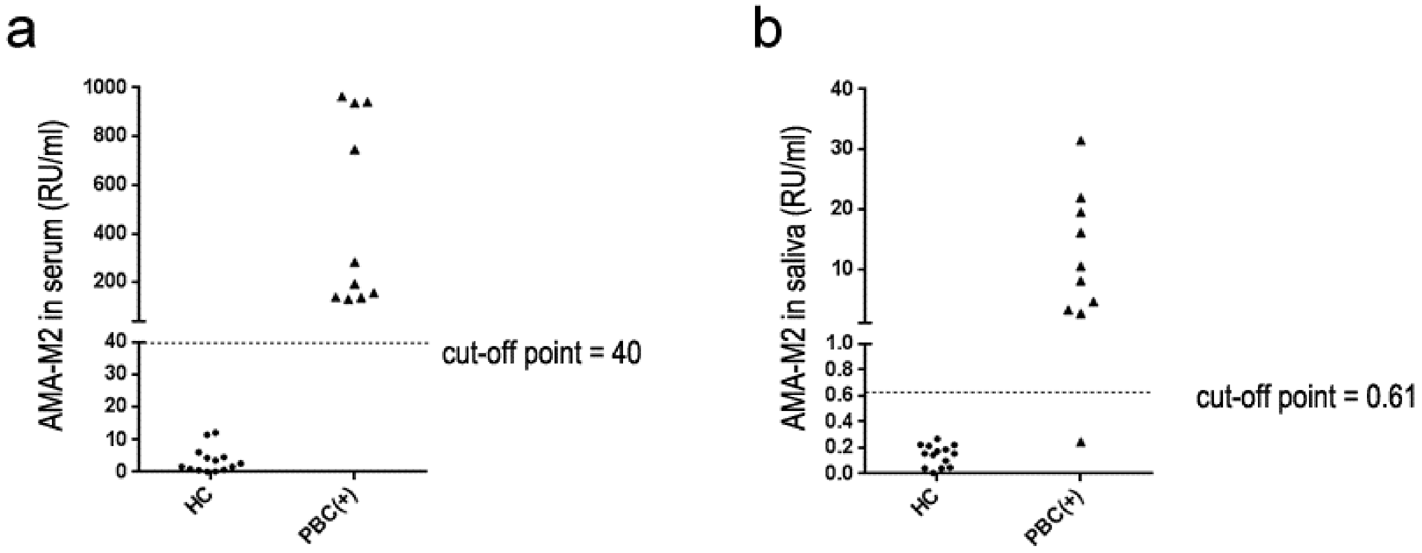

Supplementary Figure 2: AMA-M2 levels in serum and saliva of the return visit PBC patients.

The AMA-M2 level in serum **(a)** and saliva **(b)** of HCs (healthy controls, n = 14) and return visit PBC patients (n=10).

Supplementary Figure 3

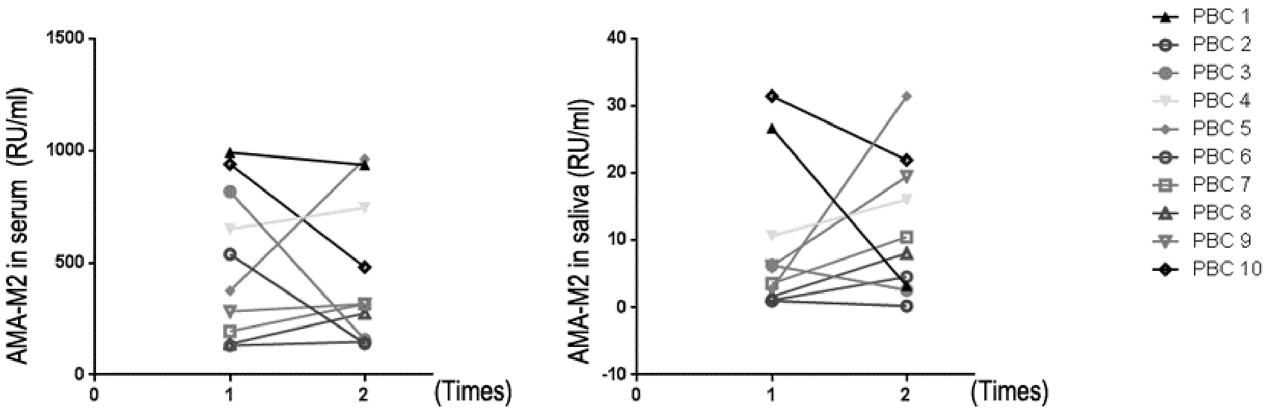

Supplementary Figure 3: The changes of serum and salivary AMA-M2 levels in two collections from the same PBC patient.

(a) Left: The changes of serum AMA-M2 levels (n=10). Right: The corresponding variation of salivary AMA-M2 levels (n=10).

Supplementary Table 1. Clinical characteristics of the individuals enrolled in the study.

	PBC patients (49 cases)	OLP patients (42 cases)
Age (year)	52.94±1.46	51.97±3.08
Gender (male/female)	7/42	16/26

Data are presented as mean ± SEM
